# Outbreak of SARS-CoV-2 Infections, Including COVID-19 Vaccine Breakthrough Infections, Associated with Large Public Gatherings — Barnstable County, Massachusetts, July 2021

**DOI:** 10.15585/mmwr.mm7031e2

**Published:** 2021-08-06

**Authors:** Catherine M. Brown, Johanna Vostok, Hillary Johnson, Meagan Burns, Radhika Gharpure, Samira Sami, Rebecca T. Sabo, Noemi Hall, Anne Foreman, Petra L. Schubert, Glen R. Gallagher, Timelia Fink, Lawrence C. Madoff, Stacey B. Gabriel, Bronwyn MacInnis, Daniel J. Park, Katherine J. Siddle, Vaira Harik, Deirdre Arvidson, Taylor Brock-Fisher, Molly Dunn, Amanda Kearns, A. Scott Laney

**Affiliations:** ^1^Massachusetts Department of Public Health; ^2^CDC COVID-19 Response Team; ^3^Broad Institute, Cambridge, Massachusetts; ^4^Barnstable County Department of Health and the Environment, Massachusetts; ^5^Community Tracing Collaborative, Commonwealth of Massachusetts.

During July 2021, 469 cases of COVID-19 associated with multiple summer events and large public gatherings in a town in Barnstable County, Massachusetts, were identified among Massachusetts residents; vaccination coverage among eligible Massachusetts residents was 69%. Approximately three quarters (346; 74%) of cases occurred in fully vaccinated persons (those who had completed a 2-dose course of mRNA vaccine [Pfizer-BioNTech or Moderna] or had received a single dose of Janssen [Johnson & Johnson] vaccine ≥14 days before exposure). Genomic sequencing of specimens from 133 patients identified the B.1.617.2 (Delta) variant of SARS-CoV-2, the virus that causes COVID-19, in 119 (89%) and the Delta AY.3 sublineage in one (1%). Overall, 274 (79%) vaccinated patients with breakthrough infection were symptomatic. Among five COVID-19 patients who were hospitalized, four were fully vaccinated; no deaths were reported. Real-time reverse transcription–polymerase chain reaction (RT-PCR) cycle threshold (Ct) values in specimens from 127 vaccinated persons with breakthrough cases were similar to those from 84 persons who were unvaccinated, not fully vaccinated, or whose vaccination status was unknown (median = 22.77 and 21.54, respectively). The Delta variant of SARS-CoV-2 is highly transmissible (*1*); vaccination is the most important strategy to prevent severe illness and death. On July 27, CDC recommended that all persons, including those who are fully vaccinated, should wear masks in indoor public settings in areas where COVID-19 transmission is high or substantial.* Findings from this investigation suggest that even jurisdictions without substantial or high COVID-19 transmission might consider expanding prevention strategies, including masking in indoor public settings regardless of vaccination status, given the potential risk of infection during attendance at large public gatherings that include travelers from many areas with differing levels of transmission.

During July 3–17, 2021, multiple summer events and large public gatherings were held in a town in Barnstable County, Massachusetts, that attracted thousands of tourists from across the United States. Beginning July 10, the Massachusetts Department of Public Health (MA DPH) received reports of an increase in COVID-19 cases among persons who reside in or recently visited Barnstable County, including in fully vaccinated persons. Persons with COVID-19 reported attending densely packed indoor and outdoor events at venues that included bars, restaurants, guest houses, and rental homes. On July 3, MA DPH had reported a 14-day average COVID-19 incidence of zero cases per 100,000 persons per day in residents of the town in Barnstable County; by July 17, the 14-day average incidence increased to 177 cases per 100,000 persons per day in residents of the town (*2*).

During July 10–26, using travel history data from the state COVID-19 surveillance system, MA DPH identified a cluster of cases among Massachusetts residents. Additional cases were identified by local health jurisdictions through case investigation. COVID-19 cases were matched with the state immunization registry. A cluster-associated case was defined as receipt of a positive SARS-CoV-2 test (nucleic acid amplification or antigen) result ≤14 days after travel to or residence in the town in Barnstable County since July 3. COVID-19 vaccine breakthrough cases were those in fully vaccinated Massachusetts residents (those with documentation from the state immunization registry of completion of COVID-19 vaccination as recommended by the Advisory Committee on Immunization Practices,^†^ ≥14 days before exposure). Specimens were submitted for whole genome sequencing^§^ to either the Massachusetts State Public Health Laboratory or the Broad Institute of the Massachusetts Institute of Technology and Harvard University. Ct values were obtained for 211 specimens tested using a noncommercial real-time RT-PCR panel for SARS-CoV-2 performed under Emergency Use Authorization at the Broad Institute Clinical Research Sequencing Platform. On July 15, MA DPH issued the first of two Epidemic Information Exchange notifications to identify additional cases among residents of U.S. jurisdictions outside Massachusetts associated with recent travel to the town in Barnstable County during July 2021. This activity was reviewed by CDC and was conducted consistent with applicable federal law and CDC policy.^¶^

By July 26, a total of 469 COVID-19 cases were identified among Massachusetts residents; dates of positive specimen collection ranged from July 6 through July 25 (Figure 1). Most cases occurred in males (85%); median age was 40 years (range = <1–76 years). Nearly one half (199; 42%) reported residence in the town in Barnstable County. Overall, 346 (74%) persons with COVID-19 reported symptoms consistent with COVID-19.** Five were hospitalized; as of July 27, no deaths were reported. One hospitalized patient (age range = 50–59 years) was not vaccinated and had multiple underlying medical conditions.^††^ Four additional, fully vaccinated patients^§§^ aged 20–70 years were also hospitalized, two of whom had underlying medical conditions. Initial genomic sequencing of specimens from 133 patients identified the Delta variant in 119 (89%) cases and the Delta AY.3 sublineage in one (1%) case; genomic sequencing was not successful for 13 (10%) specimens.

**FIGURE 1:**
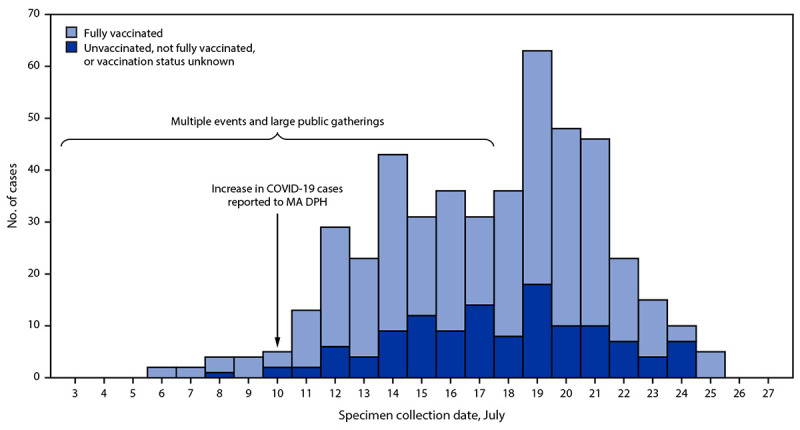
SARS-CoV-2 infections (N = 469) associated with large public gatherings, by date of specimen collection and vaccination status* — Barnstable County, Massachusetts, July 2021 **Abbreviation:** MA DPH = Massachusetts Department of Public Health. * Fully vaccinated was defined as ≥14 days after completion of state immunization registry–documented COVID-19 vaccination as recommended by the Advisory Committee on Immunization Practices.

Among the 469 cases in Massachusetts residents, 346 (74%) occurred in persons who were fully vaccinated; of these, 301 (87%) were male, with a median age of 42 years. Vaccine products received by persons experiencing breakthrough infections were Pfizer-BioNTech (159; 46%), Moderna (131; 38%), and Janssen (56; 16%); among fully vaccinated persons in the Massachusetts general population, 56% had received Pfizer-BioNTech, 38% had received Moderna, and 7% had received Janssen vaccine products. Among persons with breakthrough infection, 274 (79%) reported signs or symptoms, with the most common being cough, headache, sore throat, myalgia, and fever. Among fully vaccinated symptomatic persons, the median interval from completion of ≥14 days after the final vaccine dose to symptom onset was 86 days (range = 6–178 days). Among persons with breakthrough infection, four (1.2%) were hospitalized, and no deaths were reported. Real-time RT-PCR Ct values in specimens from 127 fully vaccinated patients (median = 22.77) were similar to those among 84 patients who were unvaccinated, not fully vaccinated, or whose vaccination status was unknown (median = 21.54) (Figure 2).

**FIGURE 2:**
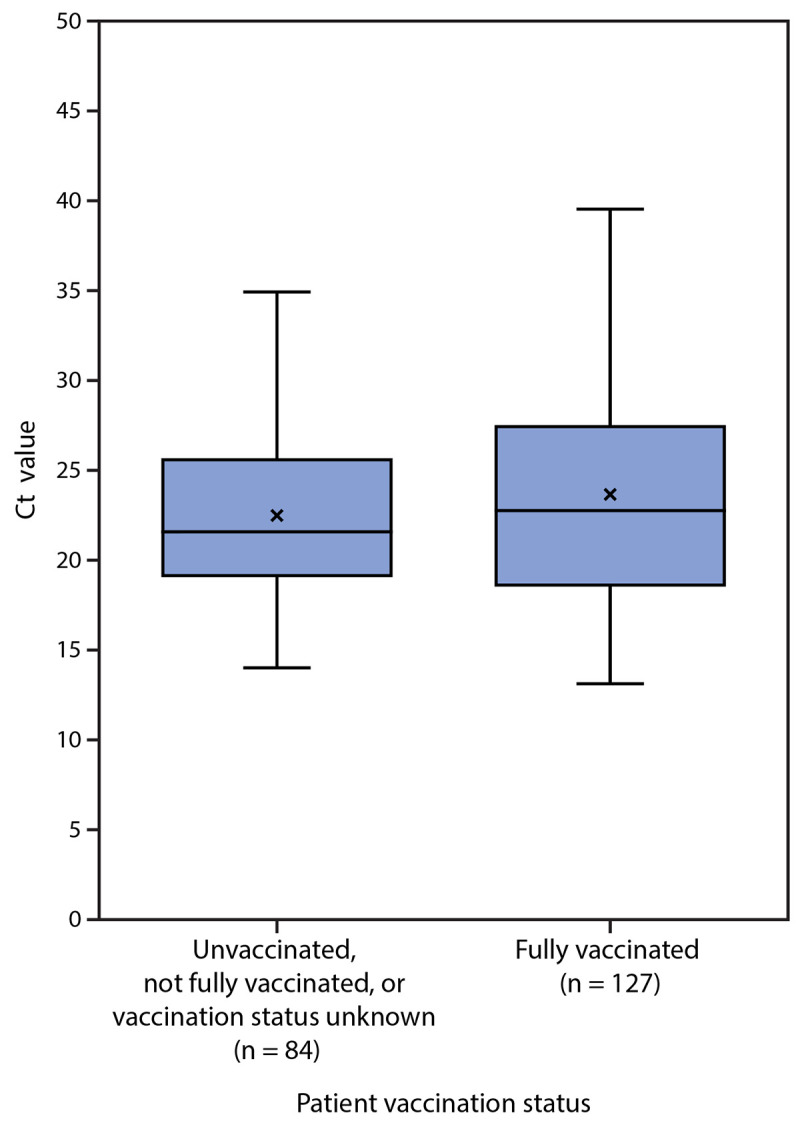
SARS-CoV-2 real-time reverse transcription–polymerase chain reaction cycle threshold values* for specimens from patients with infections associated with large public gatherings, by vaccination status^†^ — Barnstable County, Massachusetts, July 2021^§^ **Abbreviations:** Ct = cycle threshold; RT-PCR = reverse transcription–polymerase chain reaction. * Specimens were analyzed using a noncommercial real-time RT-PCR panel for SARS-CoV-2 performed under Emergency Use Authorization at the Clinical Research Sequencing Platform, Broad Institute of the Massachusetts Institute of Technology and Harvard University. ^†^ Fully vaccinated was defined as ≥14 days after completion of state immunization registry–documented COVID-19 vaccination as recommended by the Advisory Committee on Immunization Practices. ^§^ Whiskers represent minimum and maximum observations; top of box represents the third quartile (Q3), bottom represents the first quartile (Q1), and box height represents the interquartile range. Midline is the median; “x” is the mean.

Transmission mitigation measures included broadening testing recommendations for persons with travel or close contact with a cluster-associated case, irrespective of vaccination status; local recommendations for mask use in indoor settings, irrespective of vaccination status; deployment of state-funded mobile testing and vaccination units in the town in Barnstable County; and informational outreach to visitors and residents. In this tourism-focused community, the Community Tracing Collaborative^¶¶^ conducted outreach to hospitality workers, an international workforce requiring messaging in multiple languages.

The call from MA DPH for cases resulted in additional reports of cases among residents of 22 other states who had traveled to the town in Barnstable County during July 3–17, as well as reports of secondary transmission; further analyses are ongoing. As of July 3, estimated COVID-19 vaccination coverage among the eligible population in Massachusetts was 69% (*3*). Further investigations and characterization of breakthrough infections and vaccine effectiveness among this highly vaccinated population are ongoing.

## Discussion

The SARS-CoV-2 Delta variant is highly transmissible (*1*), and understanding determinants of transmission, including human behavior and vaccine effectiveness, is critical to developing prevention strategies. Multipronged prevention strategies are needed to reduce COVID-19–related morbidity and mortality (*4*). 

The findings in this report are subject to at least four limitations. First, data from this report are insufficient to draw conclusions about the effectiveness of COVID-19 vaccines against SARS-CoV-2, including the Delta variant, during this outbreak. As population-level vaccination coverage increases, vaccinated persons are likely to represent a larger proportion of COVID-19 cases. Second, asymptomatic breakthrough infections might be underrepresented because of detection bias. Third, demographics of cases likely reflect those of attendees at the public gatherings, as events were marketed to adult male participants; further study is underway to identify other population characteristics among cases, such as additional demographic characteristics and underlying health conditions including immunocompromising conditions.*** MA DPH, CDC, and affected jurisdictions are collaborating in this response; MA DPH is conducting additional case investigations, obtaining samples for genomic sequencing, and linking case information with laboratory data and vaccination history. Finally, Ct values obtained with SARS-CoV-2 qualitative RT-PCR diagnostic tests might provide a crude correlation to the amount of virus present in a sample and can also be affected by factors other than viral load.^†††^ Although the assay used in this investigation was not validated to provide quantitative results, there was no significant difference between the Ct values of samples collected from breakthrough cases and the other cases. This might mean that the viral load of vaccinated and unvaccinated persons infected with SARS-CoV-2 is also similar. However, microbiological studies are required to confirm these findings.

Event organizers and local health jurisdictions should continually assess the need for additional measures, including limiting capacity at gatherings or event postponement, based on current rates of COVID-19 transmission, population vaccination coverage, and other factors.^§§§^ On July 27, CDC released recommendations that all persons, including those who are fully vaccinated, should wear masks in indoor public settings in areas where COVID-19 transmission is high or substantial. Findings from this investigation suggest that even jurisdictions without substantial or high COVID-19 transmission might consider expanding prevention strategies, including masking in indoor public settings regardless of vaccination status, given the potential risk of infection during attendance at large public gatherings that include travelers from many areas with differing levels of transmission.

**Summary**

**What is already known about this topic?**

Variants of SARS-CoV-2 continue to emerge. The B.1.617.2 (Delta) variant is highly transmissible.

**What is added by this report?**

In July 2021, following multiple large public events in a Barnstable County, Massachusetts, town, 469 COVID-19 cases were identified among Massachusetts residents who had traveled to the town during July 3–17; 346 (74%) occurred in fully vaccinated persons. Testing identified the Delta variant in 90% of specimens from 133 patients. Cycle threshold values were similar among specimens from patients who were fully vaccinated and those who were not.

**What are the implications for public health practice?**

Jurisdictions might consider expanded prevention strategies, including universal masking in indoor public settings, particularly for large public gatherings that include travelers from many areas with differing levels of SARS-CoV-2 transmission.

## Footnotes

*
https://www.cdc.gov/coronavirus/2019-ncov/vaccines/fully-vaccinated.html

† As of May 2021, ACIP recommended that all adults aged ≥18 years receive any of the three COVID-19 vaccines available in the United States via Emergency Use Authorization from the Food and Drug Administration, including Pfizer-BioNTech, Moderna, and Janssen; persons aged ≥12 years are eligible to receive the Pfizer-BioNTech COVID-19 vaccine. Full vaccination is defined as receipt of 2 doses of the Pfizer-BioNTech or Moderna COVID-19 vaccines or 1 dose of Janssen COVID-19 vaccine ≥14 days before exposure.

§ Genomic sequencing was performed using Illumina NovaSeq using the NEB LunaScript RT ARTIC SARS-CoV-2 Kit. Novel mutations were not identified in the spike protein of the cluster-associated genomes compared with genomes collected during the same period from ongoing genomic surveillance efforts at Broad Institute. Raw and assembled genomic data are publicly available under NCBI BioProject PRJNA715749.

¶ 45 C.F.R. part 46, 21 C.F.R. part 56; 42 U.S.C. Sect.241(d); 5 U.S.C. Sect.552a; 44 U.S.C. Sect.3501 et seq.

** COVID-like symptoms were based on the Council of State and Territorial Epidemiologists surveillance case definition for COVID-19. https://ndc.services.cdc.gov/case-definitions/coronavirus-disease-2019-2020-08-05/

††
https://www.cdc.gov/coronavirus/2019-ncov/need-extra-precautions/people-with-medical-conditions.html

§§ One vaccinated, hospitalized COVID-19 patient had received the Pfizer-BioNTech vaccine and three had received the Janssen vaccine.

¶¶ The Community Tracing Collaborative is a multiorganization partnership that has supported COVID contact tracing and outbreak investigation in Massachusetts. https://www.mass.gov/info-details/learn-about-the-community-tracing-collaborative

*** A preliminary analysis matching cluster-associated COVID-19 cases with the state HIV case surveillance data identified 30 (6%) cases with verified HIV infection; all were virally suppressed, and none were hospitalized as a result of infection with SARS-CoV-2.

†††
https://www.cdc.gov/coronavirus/2019-ncov/lab/faqs.html

§§§
https://www.cdc.gov/coronavirus/2019-ncov/community/large-events/considerations-for-events-gatherings.html
